# Targeting intracellular transport combined with efficient uptake and storage significantly increases grain iron and zinc levels in rice

**DOI:** 10.1111/pbi.12943

**Published:** 2018-06-12

**Authors:** Ting‐Ying Wu, Wilhelm Gruissem, Navreet K. Bhullar

**Affiliations:** ^1^ Plant Biotechnology Department of Biology ETH Zurich Zurich Switzerland

**Keywords:** rice, iron biofortification, vacuolar iron, *NAS1*, *NRAMP3*, *FERRITIN*

## Abstract

Rice, a staple food for more than half of the world population, is an important target for iron and zinc biofortification. Current strategies mainly focus on the expression of genes for efficient uptake, long‐distance transport and storage. Targeting intracellular iron mobilization to increase grain iron levels has not been reported. Vacuole is an important cell compartment for iron storage and the NATURAL RESISTANCE ASSOCIATED MACROPHAGE PROTEIN (NRAMP) family of transporters export iron from vacuoles to cytosol when needed. We developed transgenic Nipponbare rice lines expressing *AtNRAMP3* under the control of the *UBIQUITIN* or rice embryo/aleurone‐specific *18‐kDa Oleosin (Ole18)* promoter together with *NICOTIANAMINE SYNTHASE* (*AtNAS1)* and *FERRITIN* (*PvFER*), or expressing only *AtNRAMP3* and *PvFER* together. Iron and zinc were increased close to recommended levels in polished grains of the transformed lines, with maximum levels when *AtNRAMP3*,* AtNAS1* and *PvFER* were expressed together (12.67 μg/g DW iron and 45.60 μg/g DW zinc in polished grains of line NFON16). Similar high iron and zinc levels were obtained in transgenic Indica IR64 lines expressing the *AtNRAMP3*,* AtNAS1* and *PvFER* cassette (13.65 μg/g DW iron and 48.18 μg/g DW zinc in polished grains of line IR64_1), equalling more than 90% of the recommended iron increase in rice endosperm. Our results demonstrate that targeting intracellular iron stores in combination with iron and zinc transport and endosperm storage is an effective strategy for iron biofortification. The increases achieved in polished IR64 grains are of dietary relevance for human health and a valuable nutrition trait for breeding programmes.

## Introduction

Deficiencies of essential nutrients including iron, zinc, vitamin A, vitamin B‐12, riboflavin, vitamin D and vitamin E are common and often coexist in affected populations. Iron and zinc deficiencies are widespread in developing countries but are also not uncommon in developed countries. Nearly 1.6 billion people in the world are affected by anaemia, to which iron deficiency anaemia contributes significantly (Stevens *et al*., 2013) and zinc deficiency is also reported to affect one‐third of the world's population. Diet diversification, supplementation and food fortification are among the recommended approaches to relieve these deficiencies. However, these approaches are often difficult to implement due to various regional socio‐economic factors. Furthermore, oral iron supplementation can lead to side effects including nausea and gastric pain and could increase the severity of infectious diseases, such as malaria (Oppenheimer *et al*., 1986; Sazawal *et al*., 2006). Food fortification usually causes colour and flavour changes because bioavailable iron molecules often react with other food molecules (Abbaspour *et al*., 2014; Hurrell, 2002). Biofortification for increasing iron in staple crops is therefore a promising strategy to overcome these constraints. Rice is the second most produced staple crop worldwide and consumed by more than half of the world population. Brown rice, which includes the aleurone layer of the seed coat (bran), is relatively rich in micronutrient content. However, to avoid rancidity during storage, rice is usually polished, consequently removing the nutrient‐rich bran. The polished white rice (which is mostly the endosperm of the grain) contains mainly starch and is poor in iron, zinc and other micronutrients. Most of the commercially bred rice varieties contain only around 2 μg/g iron in the endosperm (Bouis *et al*., 2011). A high content of phytate, which binds iron and zinc, in cereal grains further reduces iron and zinc bioavailability. Furthermore, it has not been possible to increase the endosperm iron concentration in rice via conventional breeding because of the limited genetic variability of endosperm iron content in the rice germplasm (Bouis *et al*., 2011). Genetic engineering approaches, however, were successful in increasing the iron concentration in polished rice grains (Vasconcelos *et al*., 2017).

The continuously developing knowledge on iron uptake, storage and translocation in rice has driven the design of iron biofortification approaches during the last few years. *FERRITIN* (*FER*)*,* a ubiquitous protein that can bind about 4500 iron atoms in a complex (Ford *et al*., 1984; Harrison *et al*., 1991), has been used to increase iron accumulation in the rice endosperm (Goto *et al*., 1999; Lucca *et al*., 2001; Oliva *et al*., 2014; Vasconcelos *et al*., 2003). Many laboratories also focused on strategies with potential to enhance iron uptake and translocation. For this, constitutive expression of *NICOTIANAMINE SYNTHASE* (*NAS*) involved in biosynthesis of deoxy‐mugineic acid (DMA), that is phytosiderophores, has been a widely used approach, with 2‐ to 4.5‐fold increased iron concentrations obtained in polished grains (Johnson *et al*., 2011; Lee *et al*., 2012; Masuda *et al*., 2009). As nicotianamine (NA) produced by NAS and mugineic acids (MAs) can also bind Zn, accompanied zinc increases in the lines overexpressing NAS are frequently reported. Among the other genes encoding iron transporters, *YELLOW STRIPE LIKE 2* (*OsYSL2*) and *IRON REGULATED TRANSPORTER 1* (*OsIRT1*) have also been transformed into rice either alone or in combination with genes related to MA synthesis and varied levels of iron increase were found in the rice grains (Ishimaru *et al*., 2010; Lee and An, 2009; Tan *et al*., 2015). YSL transporters are also known to transport zinc bound with MAs and NA. In comparison with the single‐gene strategies, constructs expressing combinations of genes involved in iron transport and uptake could achieve higher endosperm iron concentrations in rice (Boonyaves *et al*., 2016; Trijatmiko *et al*., 2016; Wirth *et al*., 2009).

Cytosolic‐free Fe concentrations are very low in plant cells because most of the iron is stored in chloroplasts, mitochondria and vacuoles in nontoxic forms and used for the synthesis of Fe‐heme or Fe‐S clusters (Finney and O'Halloran, 2003). Vacuoles have an important function in storing excess Fe and releasing it into the cytosol when the external Fe supply is suboptimal (Kim and Guerinot, 2007; Lanquar *et al*., 2010; Pich *et al*., 2001). In Arabidopsis, the vacuolar membrane located VACUOLAR IRON TRANSPORTER 1 (AtVIT1) is responsible for iron uptake into vacuoles (Kim *et al*., 2006). Oppositely, specific members of the NATURAL RESISTANCE ASSOCIATED MACROPHAGE PROTEIN (NRAMP) family mediate the efflux of iron from the vacuoles to the cytosol. NRAMP transporters localize to either intracellular vesicles, the vacuole or the plasma membrane depending on the plant species (Bereczky *et al*., 2003; Lanquar *et al*., 2005; Takahashi *et al*., 2011; Thomine *et al*., 2003). Arabidopsis AtNRAMP1, AtNRAMP3 and AtNRAMP4 mediate iron and cadmium transport, and *AtNRAMP1* is preferentially expressed in roots (Curie *et al*., 2000; Thomine *et al*., 2000). AtNRAMP3 and AtNRAMP4 are localized to the vacuolar membrane, and their genes are induced by iron deficiency (Lanquar *et al*., 2005, 2010; Thomine *et al*., 2003). Among the seven *NRAMP* homologs identified in rice, *OsNRAMP1* is involved in iron uptake (Curie *et al*., 2000) and is localized to the plasma membrane (Takahashi *et al*., 2011). Gross and colleagues (Gross *et al*., 2003) have discussed the homology between rice and Arabidopsis *NRAMP* genes in detail.

Utilizing vacuolar iron stores for increasing endosperm iron content has not been attempted to date. Here, we demonstrate that inter‐ and intracellular iron mobilization can be effectively combined for biofortification strategies in rice. To accomplish this, we expressed *AtNRAMP3* under control of either the rice *18‐kDa Oleosin* (*Ole18*) or maize *UBIQUITIN* (*Ubi*) promoters together with the endosperm‐specific expression of *PvFER* alone or in combination with the constitutive expression of *AtNAS1*. Most of the transgenic lines (background cv. Nipponbare) had significant increases of iron and zinc in both unpolished and polished grains, with maximum endosperm iron levels in lines expressing *AtNRAMP3*,* AtNAS1* and *PvFER* together. Importantly, expression of all three genes in the popular rice mega‐variety IR64 resulted in iron concentrations equalling more than 90% of the recommended iron increase in rice grains.

## Results

### Combined expression of *AtNRAMP3*,* AtNAS1* and *PvFER* increases iron and zinc concentrations in polished rice grains

**Figure 1:**
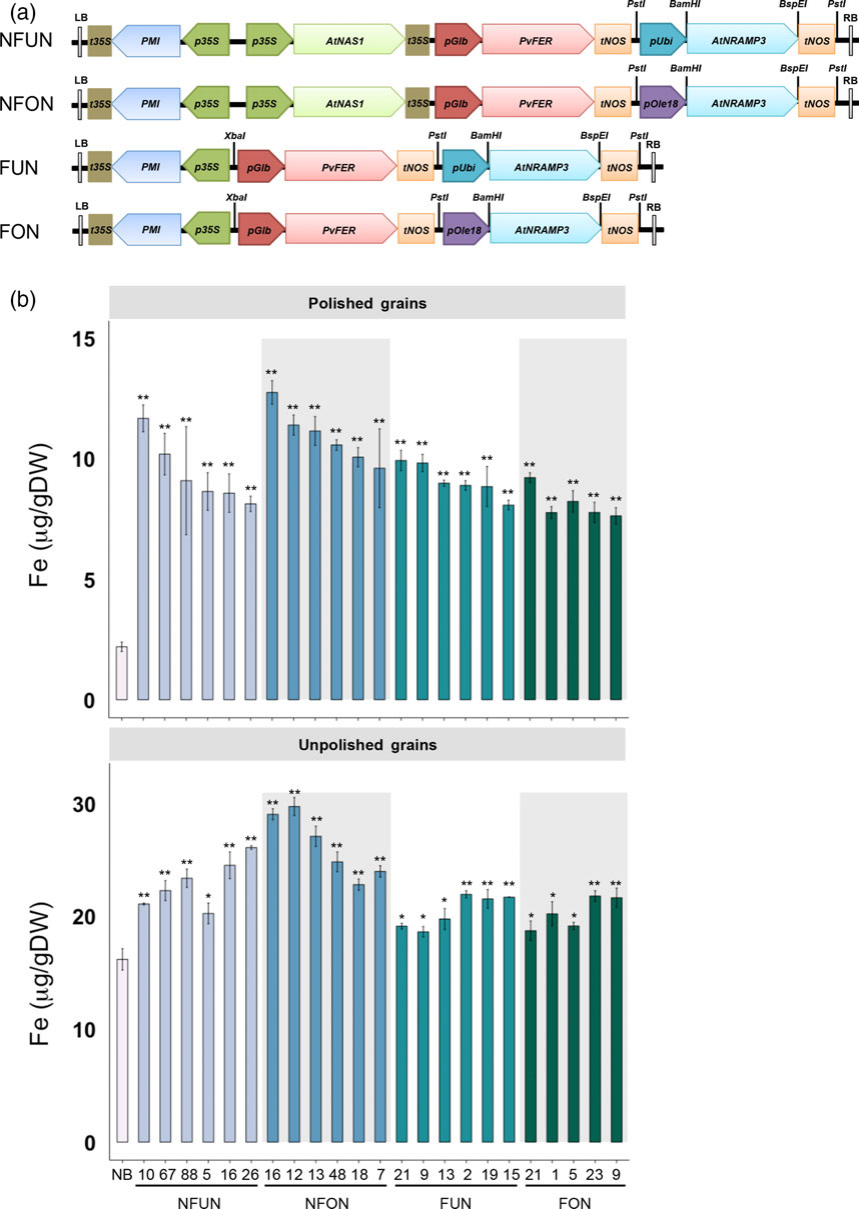
Schematic representation of the gene cassettes, and iron concentrations in polished and unpolished T3 grains. (a) The design of four constructs used for transformation. LB, T‐DNA left border; RB, T‐DNA right border; t35S, cauliflower mosaic virus (CaMV) 35S terminator; *PMI*,*PHOSPHOMANNOSE ISOMERASE* gene; *p35S*,* CaMV35S* promoter; *pUbi*, maize *UBIQUITIN* promoter; *AtNRAMP3*, Arabidopsis *NATURAL RESISTANCE‐ASSOCIATED MACROPHAGE PROTEIN 3* gene; *AtNAS1*, Arabidopsis *NICOTIANAMINE SYNTHASE 1* gene; *tNOS*,*NOPALINE SYNTHASE* terminator; *pGlb*, rice *GLOBULIN‐1* promoter; *PvFER*,* Phaseolus vulgaris FERRITIN* gene; *pOle 18*, rice *Oleosin 18* gene promoter; BamHI, BspEI, PstI and SphI represent restriction enzyme sites. (b) Iron concentrations in polished and unpolished T3 grains of lines expressing *AtNRAMP3*,* AtNAS1* and *PvFER* or *AtNRAMP3* and *PvFER* cassettes. Values are the average of three biological replicates. NB, rice cultivar Nipponbare (control); NFUN, plants expressing *pCaMV35S::At* *N**AS* *1*,*pOsGLB‐1::Pv* *F**ER* and *pZ* *m**U* *bi::At* *N**RAMP* *3*; NFON, plants expressing *pCaMV35S::At* *N**AS* *1*,*pOsGLB‐1::Pv* *F**ER* and *pO* *s**O* *le18::At* *N**RAMP* *3*; FUN, plants expressing *pOsGLB‐1::Pv* *F**ER*, and *pZ* *m**U* *bi::At* *N**RAMP* *3*; FON, plants expressing *pOsGLB‐1::Pv* *F**ER*, and *pO* *s**O* *le18::At* *N**RAMP* *3*. Black asterisks indicate statistically significantly higher values calculated using Student's *t*‐test as compared to the NB control (*P < 0.05, **P < 0.01).

**Figure 2:**
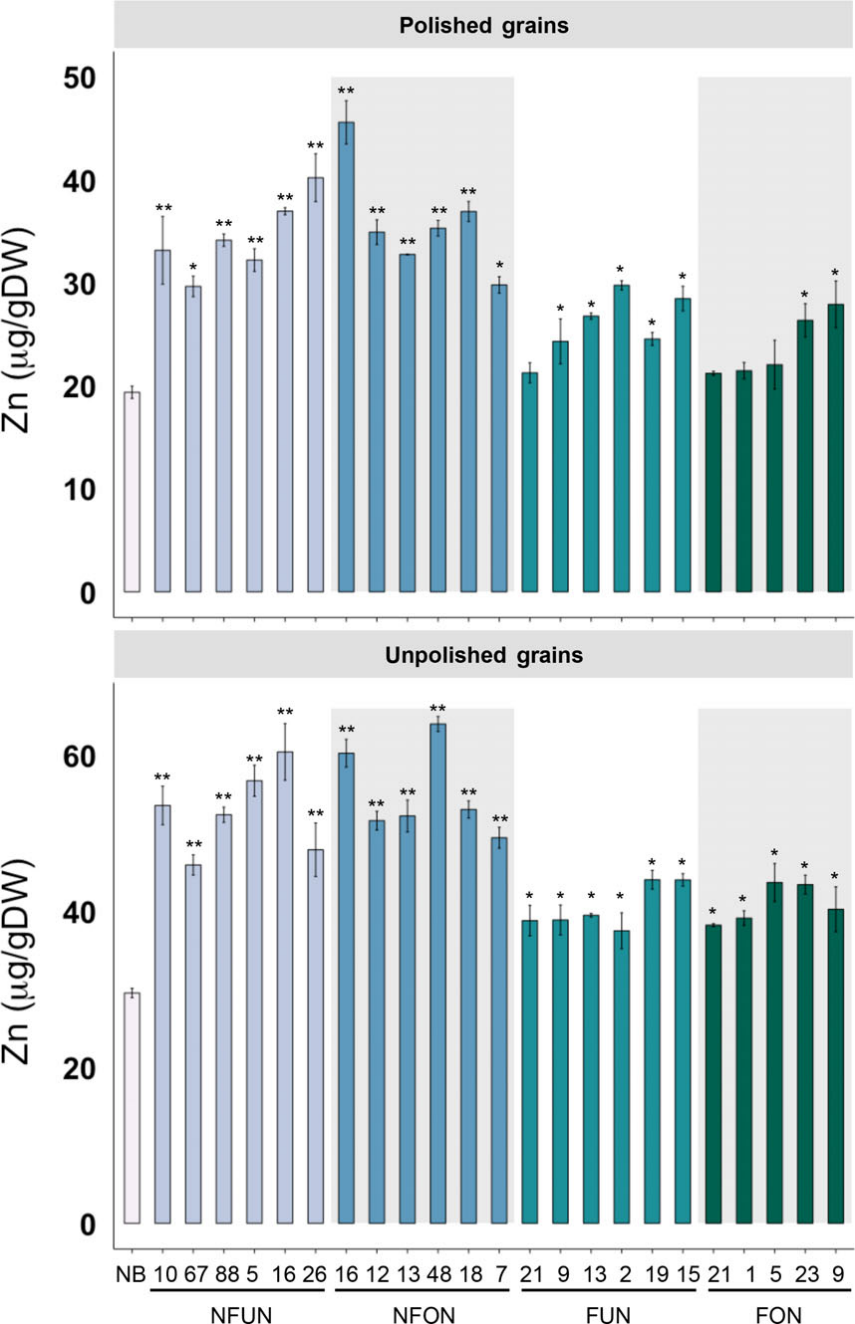
Zinc concentrations in polished and unpolished T3 grains of lines expressing *AtNRAMP3*,* AtNAS1* and *PvFER* or *AtNRAMP3* and *PvFER* alone. Values are the average of three biological replicates. Black asterisks indicate statistically significantly higher values calculated using Student's *t*‐test as compared to the NB control (*P < 0.05, **P < 0.01). See Figure 1a for the abbreviation of the gene cassettes.

We transformed the rice cultivar Nipponbare (NB) with four constructs expressing different combination of genes, abbreviated NFUN (*pCaMV35S::At* *N* *AS1*,* pOsGLB‐1::Pv* *F* *ER*,* pZm* *U* *bi::At* *N* *RAMP3*), NFON (*pCaMV35S::At* *N* *AS1*,* pOsGLB‐1::Pv* *F* *ER*,* pOs* *O* *le18::At* *N* *RAMP3*), FUN (*pOsGLB‐1::Pv* *F* *ER*,* pZm* *U* *bi::At* *N* *RAMP3*) and FON (*pOsGLB‐1::Pv* *F* *ER*,* pOs* *O* *le18::At* *N* *RAMP3*) (Figure 1a). Together, 16 independent single insertion lines for each of the NFUN, NFON and FUN constructs and 13 single insertion lines for the FON construct were selected and grown in subsequent generations (Figure S1). Based on iron concentration analysis of T2 grains, we selected five or six lines for each of the four constructs with highest endosperm iron concentration for further analysis in subsequent generations (Figure S2). All of the NFUN and FUN lines had increased iron concentrations in polished T3 grains, ranging from 8.09 to 11.69 μg/g DW, respectively, as compared to 2.12 μg/g DW iron in the NB control (Figure 1b). Similarly, NFON and FON transgenic lines had increased iron concentrations in polished grains ranging from 7.64 to 12.77 μg/g DW, with NFON 16 having a more than six‐fold higher endosperm iron concentration than the NB control (Figure 1b). In unpolished grains, NFUN and NFON lines had higher increases in iron concentrations compared to FUN and FON lines, with NFON 12 unpolished grains containing 29.75 μg/g DW iron (Figure 1b). Together, all transgenic rice lines had significantly higher iron concentrations in unpolished grains compared to NB grains. The transgene in NFON16, line with highest endosperm iron content, integrated into the first exon of a hypothetical protein encoding gene Os11 g0696100 (Figure S1b). In addition, all NFUN and NFON lines had significantly increased zinc concentrations in polished and unpolished T3 grains, ranging from 29.68 to 45.62 μg/g DW in polished grains as compared to 19.38 μg/g DW in the NB control and from 45.96 to 63.04 μg/g DW in unpolished grains compared to 29.54 μg/g DW in the NB control (Figure 2). Increases in iron and zinc concentrations were positively correlated in NFON lines. Copper concentrations were variable in some lines, while most lines had reduced manganese concentrations in the grains (Figure S3). Most transgenic plants were phenotypically similar to NB control plants and had comparable germination rates (Table S1 and Figure S4). Some of the NFON lines, however, had higher tiller numbers than NB (Table S1). With few exceptions, six grain‐quality parameters measured in the transgenic lines (grain length, grain width, chalkiness, amylose content, starch content, protein content) were similar to NB control plants (Table S2).

Together, the highest iron and zinc increases in the grains were observed in lines expressing the combination of NAS, FER and NRAMP (NFUN, NFON) as compared to the lines expressing FER and NRAMP only (FUN, FON) (Figures 1 and 2). The higher micronutrient concentrations in the NFON lines suggest that the *Ole18* promoter is equally (and in some lines even more) effective for the expression of *AtNRAMP3* compared to the *Ubi* promoter. Therefore, the *Ole18* promoter would be preferable for targeted and localized expression in biofortification strategies.

### Grain iron concentration and gene expression are positively correlated

**Figure 3:**
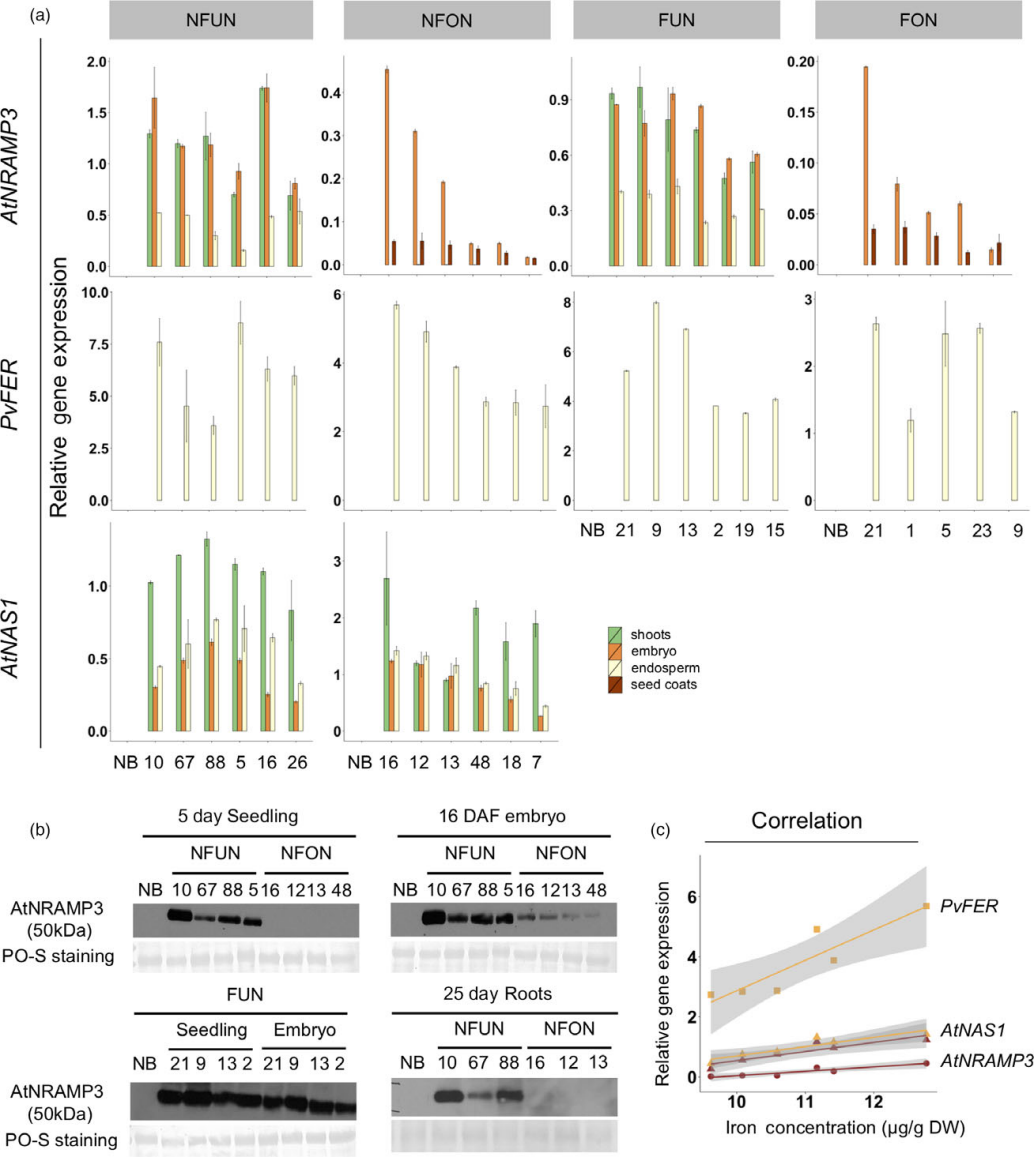
Transgene expression and AtNRAMP3 protein levels in seedlings, embryo, endosperm and seed coats. (a) qRT–PCR results of *AtNAS1*,* PvFER* and *AtNRAMP3* expression level in Nipponbare control (NB) and the selected transgenic lines. (b) Western blot analysis of AtNRAMP3 protein abundance in different tissues. Total protein extraction from NB served as a negative control. Ponceau (PO‐S) staining: loading control. Individual numbers indicate independent transgenic lines. (c) Correlation between iron content and gene expression in NFON plants (lines 16, 12, 13, 48, 18, 7). Individual lines are represented by coloured dots. See Figure 1a for the abbreviation of the gene cassettes.

The expression of the transgenes was analysed in shoots and grains (embryo, endosperm and seed coat). As expected, *AtNAS1* was expressed in all analysed tissues of NFUN and NFON lines, while expression of *PvFER* was detected only in the endosperm (Figure 3a). *AtNRAMP3* was also expressed in all the tissues but more highly in shoots and embryos of NFUN and FUN transgenic lines (Figure 3a). As expected, *AtNRAMP3* expression was restricted to the embryo and seed coat in NFON and FON lines (Figure 3a). This was further confirmed by the detection of the AtNRAMP3 protein in these specific tissues (Figure 3b). In NFON lines, transgene expression in the embryo and endosperm was positively correlated with increased iron concentration in the polished grains, indicating a synergistic action of *NAS*,* FER* and *NRAMP* (Figure 3c). As constitutive ectopic expression of *AtNRAMP3* suppresses *AtIRT1* expression in Arabidopsis plants (Thomine *et al*., 2003), we analysed the expression of the *AtIRT1* rice homolog *OsIRT1* in different tissues. Although the expression of *OsIRT1* was variable in different transgenic lines, we found no indication that *OsIRT1* expression was suppressed (Figure S5).

### NFUN and NFON lines are more tolerant to iron‐deficient growth conditions

**Figure 4:**
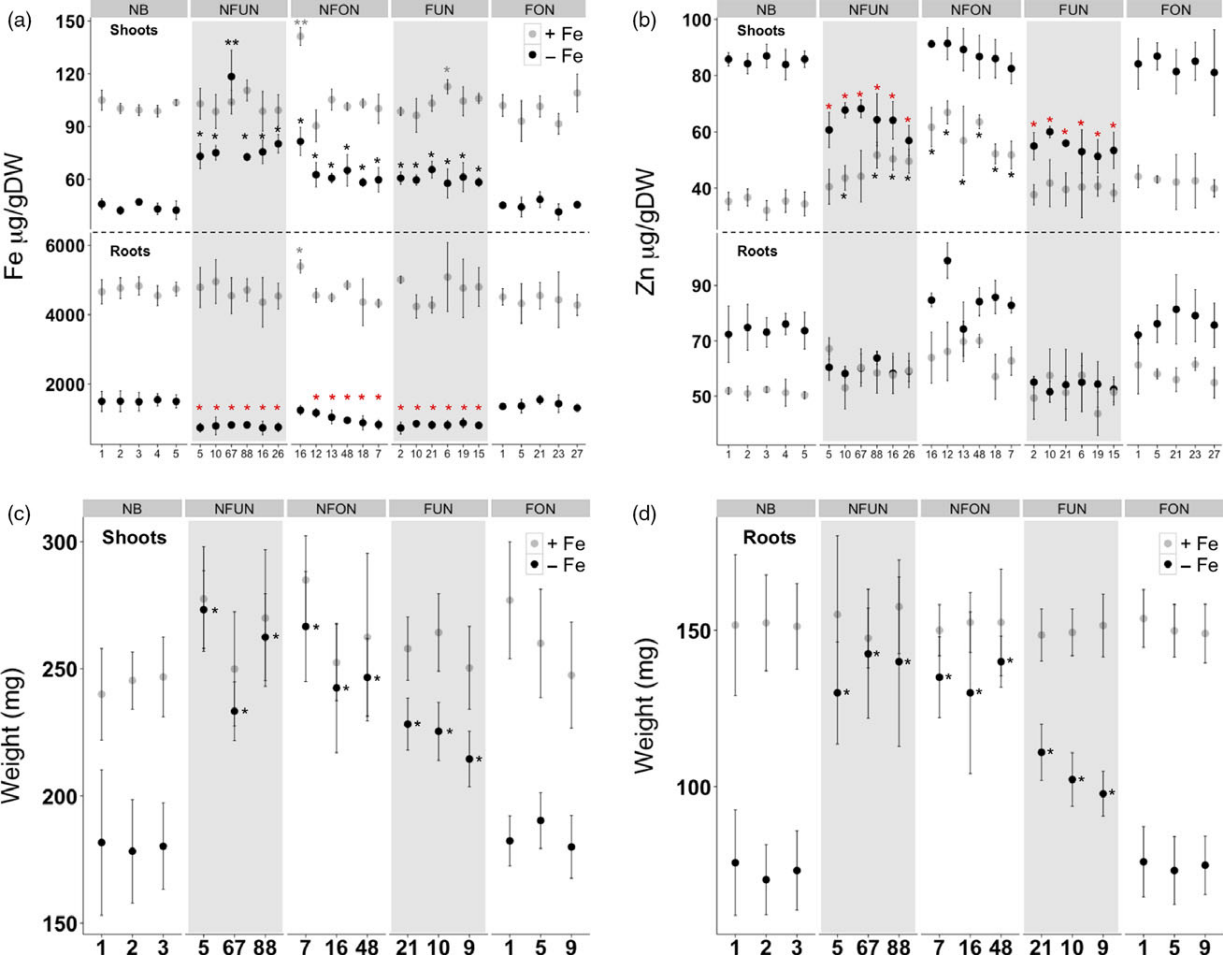
Characterization of selected transgenic lines under iron‐sufficient and iron‐deficient growth conditions. (a) Iron and (b) zinc concentrations in shoots and roots of 28‐day‐old seedlings. (c) Fresh weight of shoots and (d) roots. Samples were collected from 20 plants of each independent transgenic line, and Student's *t*‐test was used for statistical analysis. Black and red asterisks indicate statistically significantly higher and lower values as compared to the NB control (*P < 0.05, **P < 0.01), respectively. See Figure 1a for the abbreviation of the gene cassettes.

We determined the iron and zinc distribution in the shoots and roots of selected transgenic lines for each construct when grown in iron‐sufficient and iron‐deficient conditions. In iron‐sufficient conditions, most of the lines showed no significant increase in iron accumulation in the shoots and roots, except for NFON 16 that had a 1.5‐fold higher iron concentration in shoots and 1.3‐fold higher iron concentration in roots (Figure 4a). When grown in iron‐deficient conditions, most of the NFUN, NFON and FUN lines had increased iron concentrations in the shoots whereas FON lines had similar iron concentrations as NB (Figure 4a). NFUN lines had the highest iron concentration in shoots, ranging from 1.4‐fold to 1.6‐fold higher than NB. The iron concentration in roots was lower in NFUN, NFON and FUN lines, with a nearly two‐fold reduction in NFUN lines, while FON lines had similar root iron concentrations as NB roots (Figure 4a). In iron‐sufficient conditions, NFON and NFUN lines also had increased shoot zinc levels compared to NB, but root zinc levels were similar in all transgenic lines and the control (Figure 4b). In contrast, in iron‐deficient conditions shoot zinc concentrations were higher in NFON lines but 1.5‐fold and two‐fold lower in NFUN and FUN lines, respectively, compared to NB (Figure 4b). Shoot and root fresh weight was similar for transgenic plants and NB control when grown in iron‐sufficient conditions (Figure 4c,d). In iron‐deficient growth condition, however, NFUN, NFON and FUN lines had higher shoot and root fresh weight than NB, while FON transgenic plants showed no differences (Figure 4c,d). These results indicate that the expression of *AtNRAMP3* and/ or *AtNAS1* facilitates efficient iron translocation from roots to shoots, leading to increased tolerance to low‐iron condition.

Furthermore, we analysed the expression of nine endogenous genes involved in iron homeostasis in shoots and roots of plants grown under iron‐deficient and iron‐sufficient conditions. In iron‐sufficient conditions, most of the genes were expressed at similar levels in both transgenic and NB plants, but their expression was significantly different in iron‐deficient conditions (Figure S6).

### Transgenic plants do not accumulate more cadmium in grains

**Figure 5:**
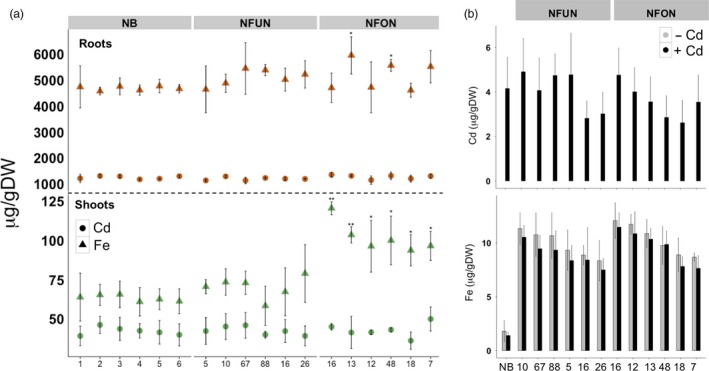
Cadmium and iron accumulation in polished T4 grains, shoots and roots of T3 plants when grown in hydroponic solution containing CdCl_2_. (a) Cadmium and iron content in shoots and roots of selected NFUN and NFON plants. Brown triangle, iron concentration in roots; brown circle, cadmium concentration in roots; green triangle, iron concentration in shoots; green circle, cadmium concentration in shoots. (b) Cadmium and iron contents in polished grains of selected NFUN and NFON plants. Black asterisks indicate statistically significantly higher values as compared to the NB control (*P < 0.05, **P < 0.01), respectively. See Figure 1a for the abbreviation of the gene cassettes.

Arabidopsis lines that overexpress *AtNRAMP3* accumulate more cadmium in leaves and roots (Thomine *et al*., 2000, 2003). We determined cadmium concentration in shoots, roots and grains of our transgenic rice plants grown in a hydroponic solution containing cadmium. The transgenic plants had similar cadmium concentrations compared to the NB control, ranging from 36.24 to 50.00 μg/g DW in shoots and from 1136.34 to 1356.58 μg/g DW in roots (Figure 5). The NFON plants had significantly higher iron concentration in the shoots as compared to NB and NFUN plants (Figure 5a). Importantly, cadmium concentrations in polished NFUN and NFON grains (2.62–4.9 μg/g DW) were comparable to NB grains (4.15 μg/g DW) (Figure 5b). Therefore, the ectopic expression of *AtNRAMP3* in rice did not increase cadmium concentrations in the grains, shoots or roots of the transgenic plants.

### IR64 lines expressing *NRAMP*,* NAS* and *FER* have dietary significant levels of iron and zinc in their grains

**Figure 6:**
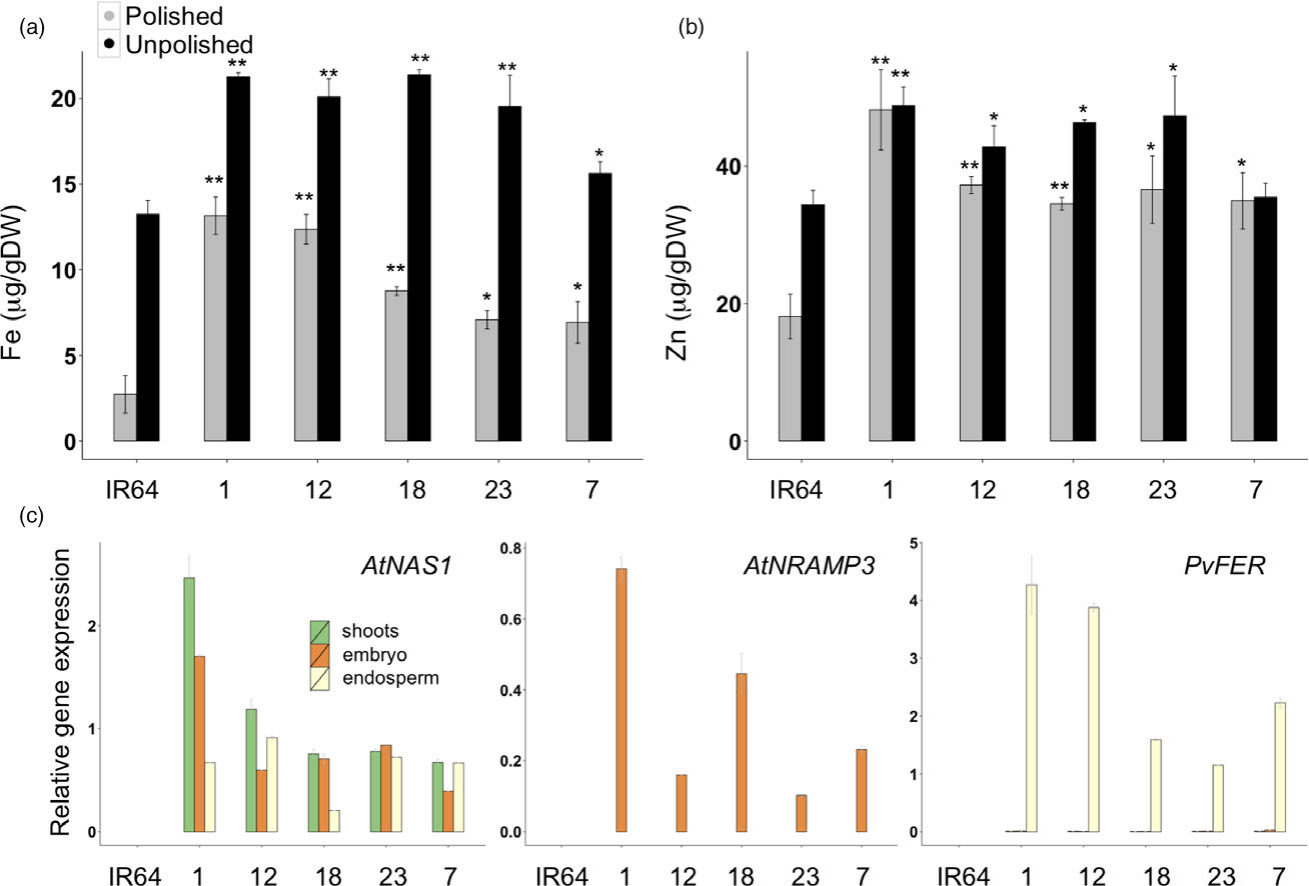
Iron and zinc concentration in polished and unpolished T2 grains of Indica cv. IR64 expressing *AtNRAMP3*,* AtNAS1* and *PvFER*. (a,b) Values are the average of three biological replicates. IR64 (control). Numbers indicate independent single insertion lines expressing *pCaMV35S::At* *N**AS* *1*,*pOsGLB‐1::Pv* *F**ER* and *pO* *s**O* *le18::At* *N**RAMP* *3*. (c) qRT–PCR results of *AtNAS1*,* PvFER* and *AtNRAMP3* expression levels in IR64 (control) and selected transgenic lines.

Based on the results with cv. Nipponbare, we transformed Indica rice cv. IR64 with the NFON construct (*pCaMV35S::At* *N* *AS1*,* pOsGLB‐1::Pv* *F* *ER*, and *pOs* *O* *le 18::At* *N* *RAMP3*; Figure 1a). Five single insertion lines were selected for further analysis (Figure S7). The IR64 transgenic plants had increased endosperm iron concentration of up to 13.65 μg/g DW in the polished grains and 21.38 μg/g DW in the unpolished grains, as compared to 2.72 and 13.24 μg/g DW iron in the polished and unpolished grains of IR64 control plants, respectively (Figure 6a). Both polished and unpolished grains of the transgenic plants also had significantly increased zinc concentrations, with the highest zinc concentration of 48.18 μg/g DW in the polished grains of IR64_1 (Figure 6b). Expression of *AtNAS1* was detected in shoots, embryo and endosperm, while *AtNRAMP3* was expressed only in the embryo and *PvFER* only in the endosperm (Figure 6c). The transgenic plants were phenotypically similar to the IR64 control in greenhouse conditions, except for IR64_12 line which had a smaller grain size (Table S3).

## Discussion

Iron biofortification in rice has been variably successful in increasing endosperm iron content. In general, strategies combining multiple genes achieve higher increases in rice endosperm iron content as compared to single‐gene strategies. To provide 30% of the dietary estimated average requirement (EAR), rice lines containing 15 μg/g DW iron and 28 μg/g DW zinc in polished grains are required (Bouis *et al*., 2011). Transgenic rice lines reported to date have iron concentrations in the range of 6–19 μg/g DW (Johnson *et al*., 2011; Masuda *et al*., 2009; Trijatmiko *et al*., 2016; Wirth *et al*., 2009). The IR64 transformed lines expressing *NAS* and *FERRITIN* reported by Trijatmiko and colleagues (Trijatmiko *et al*., 2016) grown under field conditions contained around 15 μg/g DW iron in polished grains. These increases are promising; however, the best performing lines reported contain two transgene cassette copies inserted as an inverted repeat. Transgenic plants with an inverted transgene repeat could be problematic, both from a practical and from regulatory point of view. First, two or more insertions of transgenes often result in epigenetic transgene silencing in subsequent plant generations (Kumpatla and Hall, 1998; Rajeevkumar *et al*., 2015). Second, using an inverted transgene cassette repeat in breeding programs or crop production would require a regulatory approval of uncertain, but likely negative outcome. Consequently, iron‐biofortified rice lines will be most successful when containing a single‐copy transgene cassette insertion for high‐iron concentration that is stably inherited and that can be bred efficiently into widely grown rice cultivars.

Here, we expand our biofortification strategies by combining increased iron uptake and storage with efficient intracellular iron mobilization. The expression of *AtNAS1* increases NA and DMA synthesis, both of which facilitate iron transport within the plants (Alvarez‐Fernandez *et al*., 2014; Wang *et al*., 2013 Aoyama *et al*., 2009; Rellan‐Alvarez *et al*., 2008). Additionally, we reasoned that expression of *AtNRAMP3* should remobilize iron from vacuole stores to the cytosol, which can then be transported to other tissues. By expressing *AtNRAMP3* under control of the *Ole18* promoter, we remobilized iron from the iron‐rich aleurone layer for transport to the endosperm. The endosperm‐specific expression of *PvFER* allows for effective storage of iron in the endosperm of the grains. We demonstrate that combining these genes can increase iron content more than six‐fold higher in polished seeds in two major rice cultivars, Nipponbare and IR64. The transformed IR64 lines contain up to 13.65 μg/g DW iron in polished grains, which is more than 90% of the recommended target increase of 15 μg/g DW for rice grains. The highest zinc concentration of 48 μg/g DW in transformed IR64 lines is equivalent to 170% of the recommended increase of 28 μg/g DW.

Our transgenic rice lines expressing *AtNARMP3*,* AtNAS1* and *PvFER* contain higher iron and zinc concentrations in the grains compared to lines expressing *AtNRAMP3* and *PvFER* only. In NFON lines with highest iron grain concentrations, *AtNAS1* is constitutively expressed while the *AtNRAMP3* gene and *PvFER* are regulated by the rice *Ole18* and *Glb* promoters, respectively. The *Ole18* promoter directs strong expression in the embryo and aleurone layer of rice grains primarily from 7 to 17 days after flowering (DAF), whereas the *Glb* promoter is most active between 12 and 17 DAF mostly in the endosperm (Qu le and Takaiwa, 2004). The aleurone layer provides nutrients to the developing endosperm and together with the embryo and endosperm constitutes a symplast connected by plasmodesmata that allows efficient transport and distribution of nutrients during early grain filling (Ishimaru *et al*., 2011; Thomine and Lanquar, 2011; Zhao *et al*., 2014; Zhu *et al*., 2003). Therefore, it is likely that mobilizing iron from the vacuole to the cytosol in aleurone cells and embryo scutellum could facilitate iron transport to the endosperm. This is supported by the strong correlation between iron concentrations and transgene expression in the seed tissues of NFON lines, demonstrating that targeted expression of *AtNAS1*,* PvFER* and *AtNRAMP3* is an effective strategy for increasing iron accumulation in the endosperm. Importantly, iron mobilization as a result of *AtNRAMP3* expression did not affect growth and seed germination of the transformed plants. Endosperm zinc concentrations were also higher in most of NFUN, NFON, FUN and FON lines compared to the Nipponbare control. The highest zinc concentrations were found in NFUN and NFON lines that express all transgenes, consistent with previous reports that rice lines with high grain iron concentration, particularly those expressing *NAS,* usually have higher grain zinc concentration as well (Boonyaves *et al*., 2016; Douchkov *et al*., 2005; Lee *et al*., 2009; Wirth *et al*., 2009).

Our results also show that expression of *AtNRAMP3* and/or *AtNAS1* in NFUN, NFON and FUN lines increases their tolerance to low‐iron conditions. The transgenic rice plants transport more iron from roots to shoots in iron‐deficient growth conditions, similar to transgenic tobacco plants expressing *AtNAS1* that accumulated more iron in leaves in low‐iron growth conditions and therefore were more tolerant to iron deficiency (Douchkov *et al*., 2005). High‐iron‐biofortified rice expressing *HvNAS*,* HvNAAT* and *HvIDS3* is also more tolerant to iron deficiency conditions in hydroponic culture and calcareous soil (Masuda *et al*., 2013). Expression of *AtNRAMP3* alone can rescue the hypersensitive iron starvation phenotype of the Arabidopsis *atnr3nr4* double mutant (Lanquar *et al*., 2005). Together, the tolerance of our transgenic lines to iron deficiency can be explained by the expression of *AtNAS1* and increased cytosolic iron availability in vegetative tissues as the result of *AtNRAMP3* expression. Similar to Arabidopsis plants expressing *AtNRAMP3* that have reduced root and shoot zinc and manganese levels in iron deficiency conditions (Thomine *et al*., 2003), we also found reduced zinc concentrations in NFUN and FUN plants compared to the Nipponbare control in iron deficiency conditions. However, NFON and FON plants did not differ significantly from the Nipponbare control plants in their shoot and root zinc concentrations when grown in the same conditions. This is likely due to a localized expression of *AtNRAMP3* in the NFON and FON plants.

Metal transporters often have a broader specificity; that is, transporters facilitating the uptake of essential metal ions including Fe, Zn, Cu and Mn may also transport heavy metal ions including cadmium (Cd). Particularly, Zn‐specific transporters can cotransport Cd, and the NRAMP transporter family is known to transport Cd as well (DalCorso *et al*., 2010; Olsen and Palmgren, 2014; Papoyan *et al*., 2007). Thus, it is possible that expression of iron transporters to develop iron‐biofortified rice may also cause the accumulation of nondesired toxic metals that could be detrimental to human health. The results of hydroponics experiments demonstrate that our transgenic rice lines did not have higher Cd concentrations in polished grains compared to the Nipponbare control. This supports other studies that also reported no increases in the Cd accumulation in grains of high‐iron‐biofortified rice (Masuda *et al*., 2012; Trijatmiko *et al*., 2016).

Together, the combinatorial and targeted expression of *AtNAS1*,* AtNRAMP3* and *PvFER* is a novel and promising strategy for achieving significant iron and zinc increases in polished rice grains. The IR64 lines with highest iron concentration we report here are now important candidates for the development of agronomically robust rice mega‐varieties with improved nutritional benefits for human health.

## Experimental procedures

### DNA construct, rice transformation and plant growth conditions

The full‐length *AtNRAMP3* genomic sequence was amplified using a forward primer containing a *BamHI* site and a reverse primer with a *BspEI* site (Table S4). The fragment was subsequently inserted into the *BamHI*‐ and *BspEI*‐digested *OsNAS2‐PbskII(‐)* vector (Singh *et al*., 2017a,b) to generate *pUbi::AtNRAMP3::tNOS* fragment. In parallel, the *PstI* site in the *Ubi* promoter contained in the *OsNAS2‐PbskII(‐)* construct was changed by a point mutation to use the site for subsequent cloning steps. The *pCAMBIA‐1300PMI‐NASFER* vector containing the *AtNAS1* and *PvFER* genes was cut at the *PstI* site and the *pUbi::AtNRAMP3::tNOS* fragment was inserted to generate the NFUN cassette. Similarly, *pOle18* promoter sequence was amplified using a forward primer containing *PstI* and *SphI* sites, and a reverse primer containing a *BamHI* site, and then inserted into *pUbi::AtNRAMP3‐PbskII(‐)* digested with *SphI* and *BamHI* to generate *pOle18::AtNRAMP3::tNOS*. The *pCAMBIA‐1300PMI‐NASFER* vector was then digested at the *PstI* site and the *pOle18::AtNRAMP3::tNOS* fragment was inserted to generate the NFON cassette. For the FUN and FON constructs, *pCAMBIA‐1300PMI‐NASFER* was digested at the *XbaI* and *PstI* sites to obtain the *pGlb::PvFERRITIN::tNOS* fragment, which was subsequently inserted into *pCAMBIA‐1300PMI* to generate the *pCAMBIA‐1300PMI‐FER* vector. The *pCAMBIA‐1300PMI‐FER* vector was cut at the *PstI* site, and the *pUbi::AtNRAMP3::tNOS* or *pOle18::AtNRAMP3::tNOS* fragments were inserted to generate the FUN or FON cassettes, respectively. These four vectors were transformed into *Oryza sativa ssp. Japonica cv*. Nipponbare, and NFON was additionally transformed into *Indica* IR64 using *Agrobacterium tumefaciens* strain EHA105 (Hood *et al*., 1993). Transformation, selection and regeneration followed an established protocol (Nishimura *et al*., 2006). Candidate transformants were first screened for the presence of *AtNRAMP3*,* AtNAS1* and *PvFER* using PCR. Southern blot hybridization using digoxigenin (DIG) labelling was performed using *PmlI*‐digested genomic DNA isolated from the transgenic lines to select lines with single‐copy cassette insertions. The PCR‐amplified *PMI* DNA fragment was used as a probe to detect the transgene cassette. The primer sequences used for cloning, sequence verification and PCR are provided in Table S4. Regions flanking the transgene in NFON16 were amplified using TAIL‐PCR (Liu and Whittier, 1995). In brief, three consecutive PCRs were performed using three arbitrary primers and nested specific primers (Table S5). All TAIL‐PCRs were performed in 20 μL reaction volumes. The first TAIL‐PCR was performed using approximately 100 ng template DNA. The second PCR was performed using a 1:40 dilution of the preceding PCR and the third PCR using 1 μL of the PCR product of the second TAIL‐PCR. Cycling conditions were as specified by Liu and Whittier (1995). PCR products were sequenced by Microsynth AG (Balgach, Switzerland).

The plants were grown in commercial soil ‘ETH Soil mixture 1' – Ricoter composition (25% field soil, sterile, 0‐15 mm; 25% bark mulch; 20% sand, washed, 0–2 mm and 30% white peat, 7–20 mm) under greenhouse conditions in 80% humidity at 30 °C with 12‐h light and 60% humidity at 22 °C with 12‐h dark. Quantification of divalent metal ions and transgene expression was conducted using T2 and T3 grains.

### Iron deficiency and cadmium excess treatment in hydroponic culture

Transgenic and Nipponbare (NB) seeds were germinated *in vitro* for 5 days in Petri dishes on a H_2_O‐moistened filter paper and subsequently transferred into containers (with tip holes) containing 400 mL hydroponic solutions for 7 days. Solutions for hydroponic growth were prepared according to established protocols (Boonyaves *et al*., 2016; Kobayashi *et al*., 2005), that is, using 0.70 mm K_2_SO_4_, 0.10 mm KCl, 0.10 mm KH_2_PO_4_, 2.0 mm Ca(NO_3_)_2_, 0.50 mm MgSO_4_, 10 μm H_3_BO_3_, 0.50 μm MnSO_4_, 0.20 μm CuSO_4_, 0.01 μm (NH4)_6_Mo_7_O_24_, and 0.5 μm ZnSO_4_, with different iron concentrations added as Fe(III)‐EDTA according to the treatment (normal condition: 100 μm; iron‐deficient condition: 10 μm). For the iron‐deficient condition, plants were grown in hydroponic solutions containing 10 μm Fe(III)‐EDTA for 14 days. Plants grown in hydroponic solutions containing 100 μm Fe(III)‐EDTA served as control. Solutions were changed every 2 days to avoid any precipitation and contamination. In case of Cd excess treatment, plants were grown in hydroponic solutions containing 50 μm CdCl_2_ and shoot and root samples were collected from 21‐day‐old seedlings. To analyse grain Cd concentrations, plants were grown until maturity in a hydroponic solution containing 10 μm CdCl_2_ in greenhouse conditions in 80% humidity at 30 °C with 12‐h light and 60% humidity at 22 °C with 12‐h dark.

### Metal ion measurements

Grain samples were dehusked to obtain unpolished brown grains. The dehusked grains were processed using a grain polisher (Kett grain polisher ‘Pearlest’, Kett Electric Laboratory, Tokyo, Japan) for 1 min. Shoot and root samples from hydroponic culture were dried at 60 °C for 5 days. Two hundred milligrams of each ground grains sample and 50–100 mg of root or shoot samples were boiled in 15 mL of 65% v/v HNO_3_ solution at 120 °C for 90 min. Three millilitres of 30% v/v H_2_O_2_ was subsequently added and continuously boiled at 120 °C for 90 min. Metal concentrations were determined using inductively coupled plasma–optical emission spectroscopy (ICP‐OES) (Varian Vista‐MPX CCD Simultaneous ICP‐OES). The wavelength used for iron, zinc, manganese and copper were 238.204, 213.857, 257.610 and 324.754 nm, respectively. The National Institute of Standards and Technology (NIST) rice flour standard 1658a was treated and analysed in the same manner and used as internal control for every measurement. Data were analysed using Student's *t*‐test. The criteria of *P* < 0.05 and *P* < 0.01 were used to determine statistically significant differences among the tested lines and the control.

### RNA extraction, cDNA synthesis and quantitative real‐time PCR

Total RNA was extracted from roots and shoots of 5‐day‐old T3 generation seedlings using Trizol^®^ reagent (Invitrogen of Thermo Fischer Scientific Inc., Massachusetts (MA), USA). To obtain total endosperm, embryo and seed coat RNA, whole seed coats, endosperm and embryo were separated manually from T3 rice grains at 16 DAF. Extraction buffer containing 0.15m NaCl and 1% sarcosyl sarcosyl (N‐Lauroyl sarcosinate sodium) was added to the ground samples followed by purification with 8m guanidine hydrochloride buffer. The RNA was treated with DNase I (Thermo Fisher Scientific Inc., Massachusetts (MA), USA) to remove genomic DNA contamination. First‐strand cDNA was synthesized using the RevertAid™ first‐strand cDNA synthesis kit (Thermo Fisher Scientific Inc.). Quantitative RT–PCR was performed as previously described (Liu *et al*., 2011). In brief, qRT–PCR was performed in a 7500 Real‐Time PCR System using the SYBR Green RT–PCR reagent kit following the manufacturer's protocol (Applied Biosystems, Carlsbad, CA). Each reaction was run in triplicates in a volume of 20 μL with an initial denaturation step at 95 °C for 10 min, followed by 40 cycles of 95 °C for 15 s and 60 °C for 60 s. Data were analysed according to the manufacturer's instructions using the 7500 System SDS Software v1.4 (Applied Biosystems). The expression level of genes of interest was normalized to the expression of rice *UBIQUITIN 5* (*OsUBQ5)*.

### Protein extraction and Western blotting analysis

Protein extraction and Western hybridization were carried out as described previously (Svozil *et al*., 2015). In brief, the frozen plant material was ground into a fine powder and proteins were extracted by incubation with SDS buffer containing 4% SDS, 40 mm Tris‐base, 5 mm MgCl_2_ and 2x protease inhibitor mix (Roche Roche AG, Basel, Switzerland) for 20 min at room temperature (RT). Cell debris was removed by centrifugation at RT for 10 min at 16 000× *g*. The supernatant was stored at −80 °C for further use. For the Western blots, 30 μg of leaf extract and 50 μg of embryo extract was separated using SDS–PAGE on 10% SDS gels. After blotting, the nitrocellulose membranes were probed with an anti‐AtNRAMP3 (1:1000) antibody for 1 h at RT and then followed by an anti‐rabbit (1:5000, Roche) antibody for 1 h at RT. After detection of the immunofluorescence signal, the membranes were stained with Ponceau Red for 10 min.

### Germination rate test

Thirty seeds of each tested line were imbibed in deionized water at RT for 2 days in the dark and then transferred to Petri dishes containing water‐saturated filter paper for germination. Germination rates were scored every day. Three replications were performed using 30 seeds each time. The test was performed according to a published protocol (Cheng *et al*., 2013; Yang *et al*., 2012) with the above modifications.

## Conflict of interest

The authors declare that they have no conflicts of interest.

## Author contributions

NKB conceived the study, NKB and TYW designed the experiments, TYW performed the experiments, TYW, WG and NKB interpreted the data, TYW and NKB wrote the manuscript, and WG and NKB edited the manuscript. All authors have read and approved the final manuscript.

## Supporting information


**Figure S1** (a) Characterization of transgenic lines in T0 generation using southern hybridization. Southern hybridization analysis was performed using PMI as a probe. Numbers indicate independent transformed lines with potential single insertion of the transgene cassette. (b) Flanking sequences and position of T‐DNA integration in NFON 16. The transgene cassette integrated in the first exon of Os11 g0696100 (encoding for a hypothetical protein). Red and grey sections indicate exon and intron, respectively. Black section represents nucleotides deleted during transgene integration.
**Figure S2** Iron concentration in the polished T2 grains in all the single insertion lines carrying NFUN, NFON, FUN and FON constructs.
**Figure S3** Manganese and copper concentrations in polished and unpolished T3 grains expressing *AtNRAMP3*,* AtNAS1* and *PvFER* or *AtNRAMP3* and *PvFER* cassettes.
**Figure S4** Germination rate analysis of seeds from selected transgenic plants compared to the Nipponbare control.
**Figure S5** Endogenous *OsIRT1* expression in shoots, roots, embryo and endosperm of selected transgenic plants.
**Figure S6** Expression analysis of iron homeostasis related genes.
**Figure S7** Characterization of IR64 transgenic lines in T0 generation using southern hybridization and iron concentration in the polished T1 grains in the single insertion lines.
**Table S1** Phenotypic assessment of selected transgenic lines in T2 generation grown in the greenhouse condition
**Table S2** Grain quality assessment of selected transformed plants (T3) carrying NFUN, FNON, FUN and FON constructs as compared to the Nipponbare control
**Table S3** Phenotypic assessment of selected transgenic lines (cv. IR64 background) from the T1 generation grown in greenhouse conditions
**Table S4** Primer sequences used for sequence validation, cloning and qRT–PCR analysis
**Table S5** Primer sequences used for TAIL‐PCR analysis on NFON16
