# Supplementary material for: Targeting intracellular transport combined with efficient uptake and storage significantly increases grain iron and zinc levels in rice
Source: Plant Biotechnol J. 2018 Jun 12;17(1):9–20. doi: 10.1111/pbi.12943 (PMC6330537; doi:10.1111/pbi.12943)
Supplement: Supplementary file 1 — Figure S1 (a) Characterization of transgenic lines in T0 generation using southern hybridization. Southern hybridization analysis was performed using PMI as a probe. Numbers indicate independent transformed lines with potential single insertion of the transgene cassette. (b) Flanking sequences and position of T‐DNA integration in NFON 16. The transgene cassette integrated in the first exon of Os11 g0696100 (encoding for a hypothetical protein). Red and grey sections indicate exon and intron, respectively. Black section represents nucleotides deleted during transgene integration. Figure S2 Iron concentration in the polished T2 grains in all the single insertion lines carrying NFUN, NFON, FUN and FON constructs. Figure S3 Manganese and copper concentrations in polished and unpolished T3 grains expressing AtNRAMP3, AtNAS1 and PvFER or AtNRAMP3 and PvFER cassettes. Figure S4 Germination rate analysis of seeds from selected transgenic plants compared to the Nipponbare control. Figure S5 Endogenous OsIRT1 expression in shoots, roots, embryo and endosperm of selected transgenic plants. Figure S6 Expression analysis of iron homeostasis related genes. Figure S7 Characterization of IR64 transgenic lines in T0 generation using southern hybridization and iron concentration in the polished T1 grains in the single insertion lines. Table S1 Phenotypic assessment of selected transgenic lines in T2 generation grown in the greenhouse condition Table S2 Grain quality assessment of selected transformed plants (T3) carrying NFUN, FNON, FUN and FON constructs as compared to the Nipponbare control Table S3 Phenotypic assessment of selected transgenic lines (cv. IR64 background) from the T1 generation grown in greenhouse conditions Table S4 Primer sequences used for sequence validation, cloning and qRT–PCR analysis Table S5 Primer sequences used for TAIL‐PCR analysis on NFON16 [file PBI-17-9-s001.docx]

**Supporting information**

**Figure S1 (a) Characterization of transgenic lines in T0 generation using southern hybridization.** Southern hybridization analysis was performed using PMI as a probe. Numbers indicate independent transformed lines with potential single insertion of the transgene cassette. **(b) Flanking sequences and position of T-DNA integration in NFON 16.** The transgene cassette integrated in the first exon of Os11g0696100 (encoding for a hypothetical protein). Red and grey sections indicate exon and intron, respectively. Black section represents nucleotides deleted during transgene integration.

**
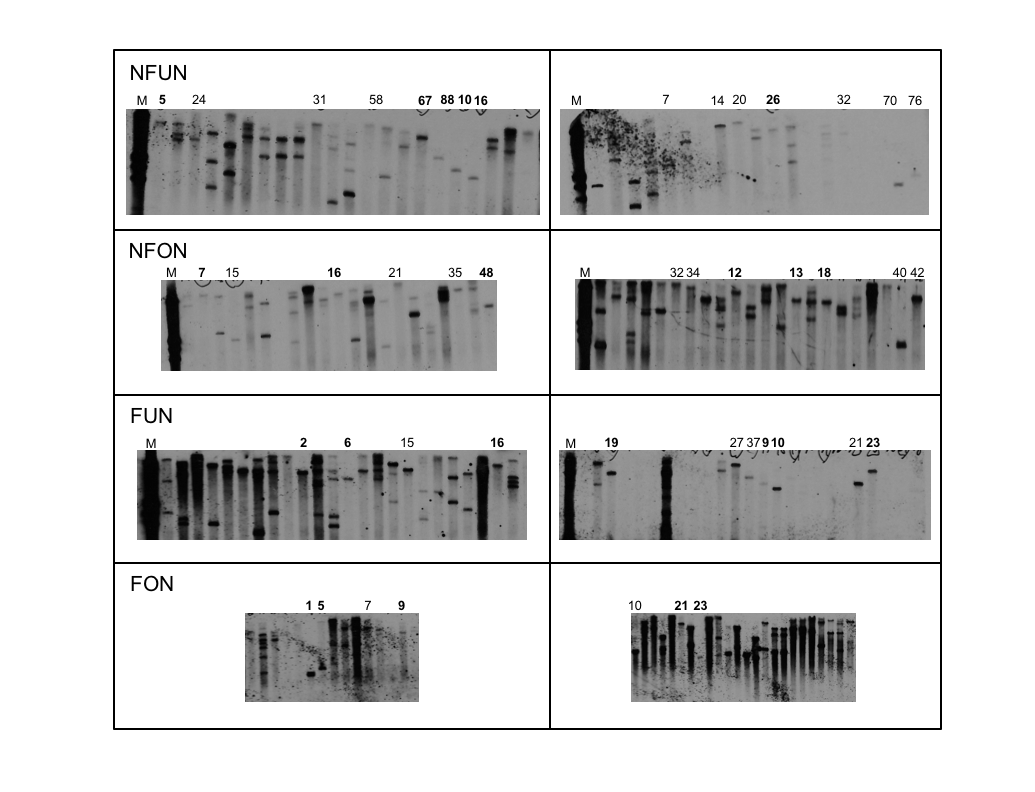
**

**(b)**

**Figure S2 Iron concentration in the polished T2 grains in all the single insertion lines carrying NFUN, NFON, FUN and FON constructs.** Values are the average of two technical replicates. The bars for each line represent three individual plants. Arrows indicate the selected plants from each line for further analysis in next generation and II indicate second batch of transformation; NB, rice cultivar Nipponbare (control). See Figure 1a for the abbreviation of the gene cassettes.

**
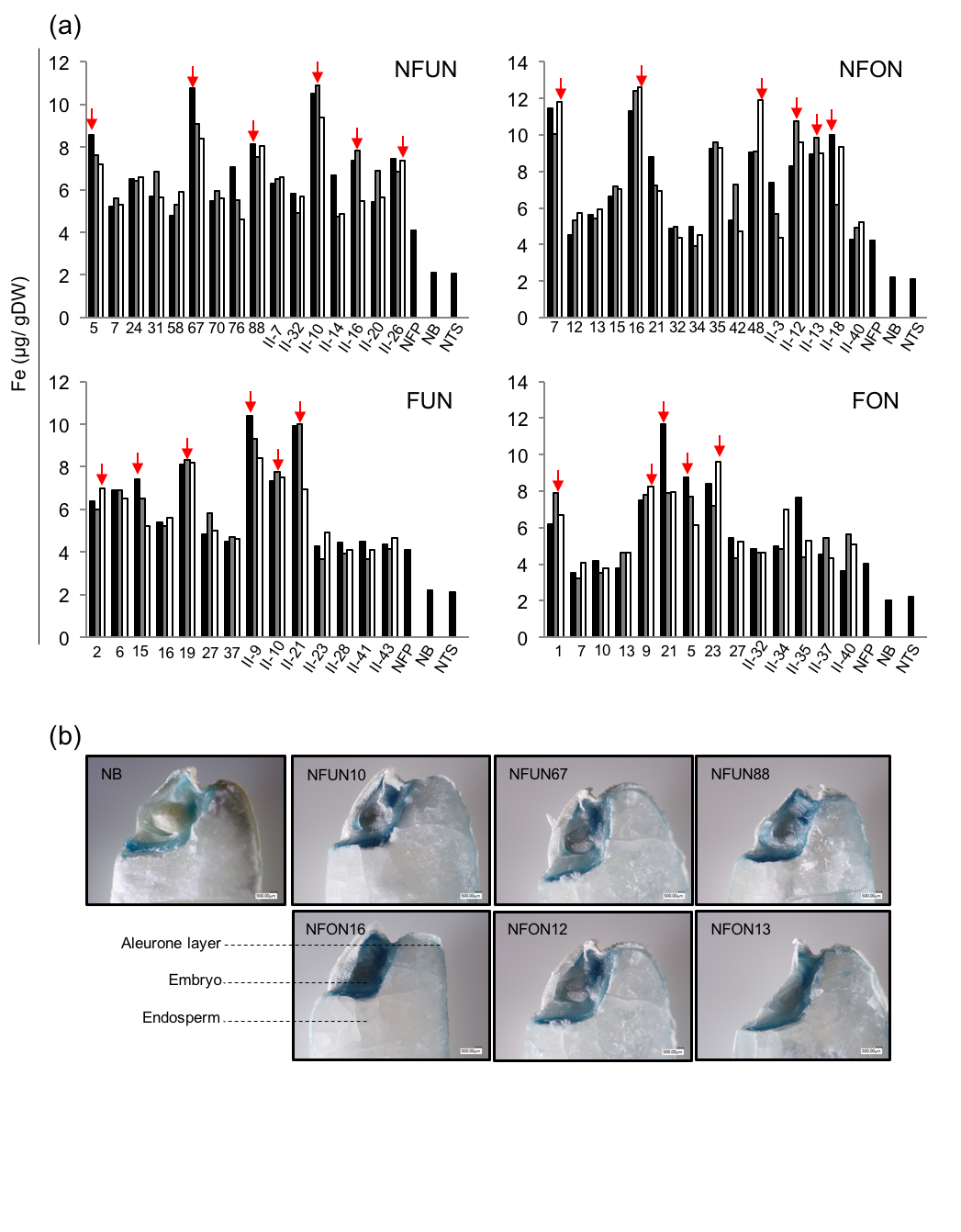
**

**Figure S3 Manganese and copper concentrations in polished and unpolished T3 grains expressing *AtNRAMP3*, *AtNAS1* and *PvFER* or *AtNRAMP3* and *PvFER* cassettes.** Values are the average of three biological replicates. NB, Nipponbare rice control. Black asterisks indicate statistically significant higher values calculated using Student’s T-Test as compared to NB control (*p<0.05, **p<0.01). See Figure 1a for the abbreviation of the gene cassettes. **
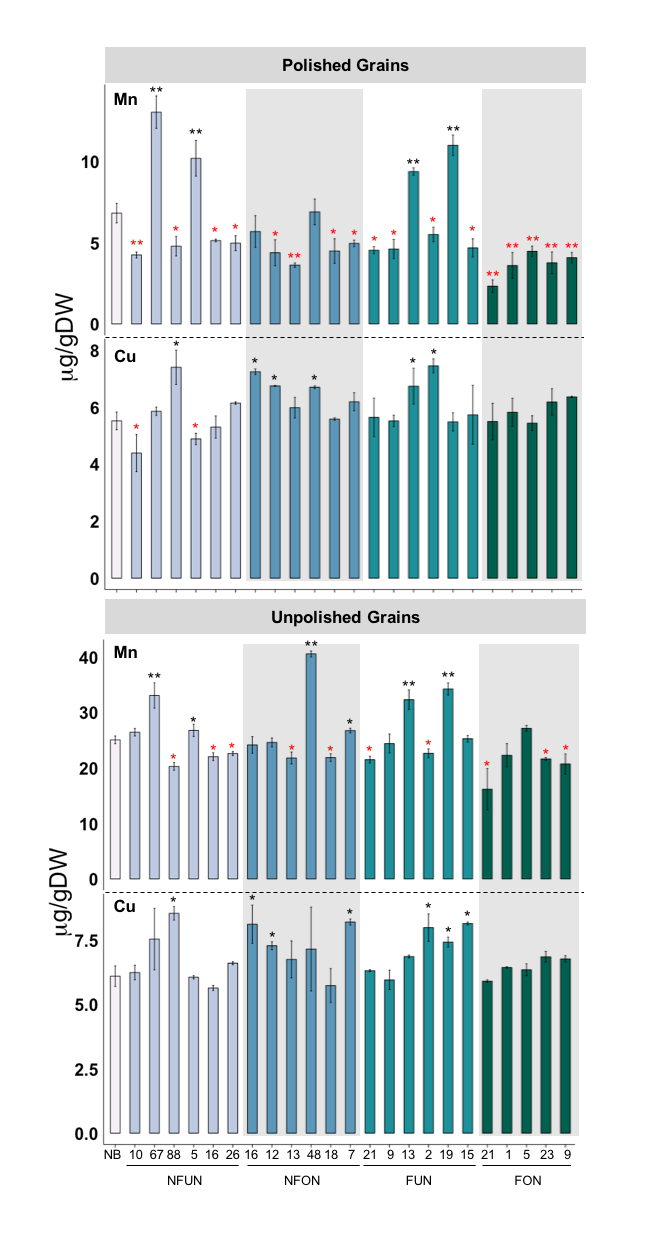
**

**Figure S4 Germination rate analysis of seeds from selected transgenic plants compared to the Nipponbare control.** Germination rate of seeds from (a) NFUN, (b) NFON, (*C*) FUN and (*D*) FON plants at 24, 48 and 72 hours. Thirty seeds were tested in each experiment and 3 individual experiments were conducted. See Figure 1a for the abbreviation of the gene cassettes.

**
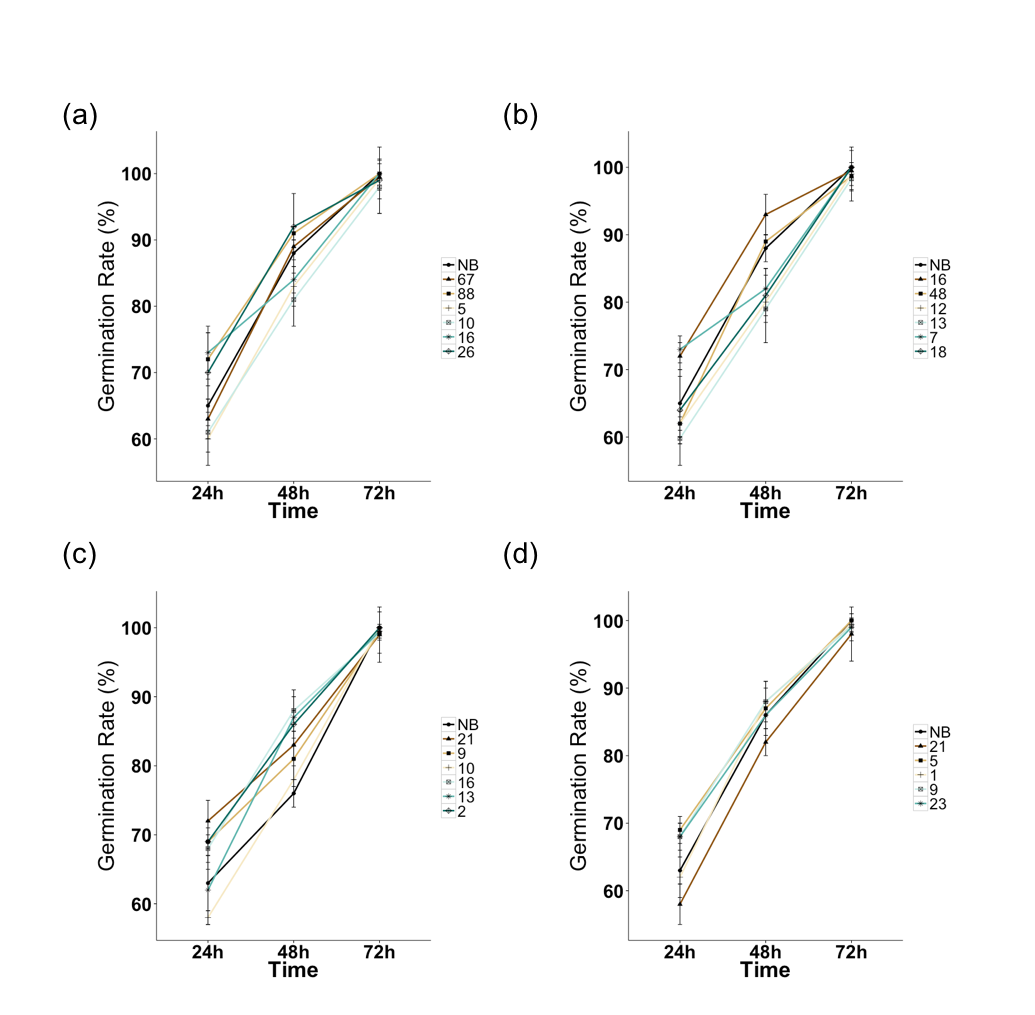
**

**Figure S5 Endogenous *OsIRT1* expression in shoots, roots, embryo and endosperm of selected transgenic plants.** *OsIRT1* expression determined by qRT-PCR in shoots, roots, embryo and endosperm of transgenic plants expressing the (a) NFUN and (b) NFON cassettes, and in embryo and endosperm of transgenic plants expressing the (c) FUN and (d) FON cassettes. Numbers on the figure indicate independent transgenic lines. Expression values were normalized to the expression values of the rice *UBIQUITIN5* (*OsUBQ5*) gene. See Figure 1a for the abbreviation of the gene cassettes.

**
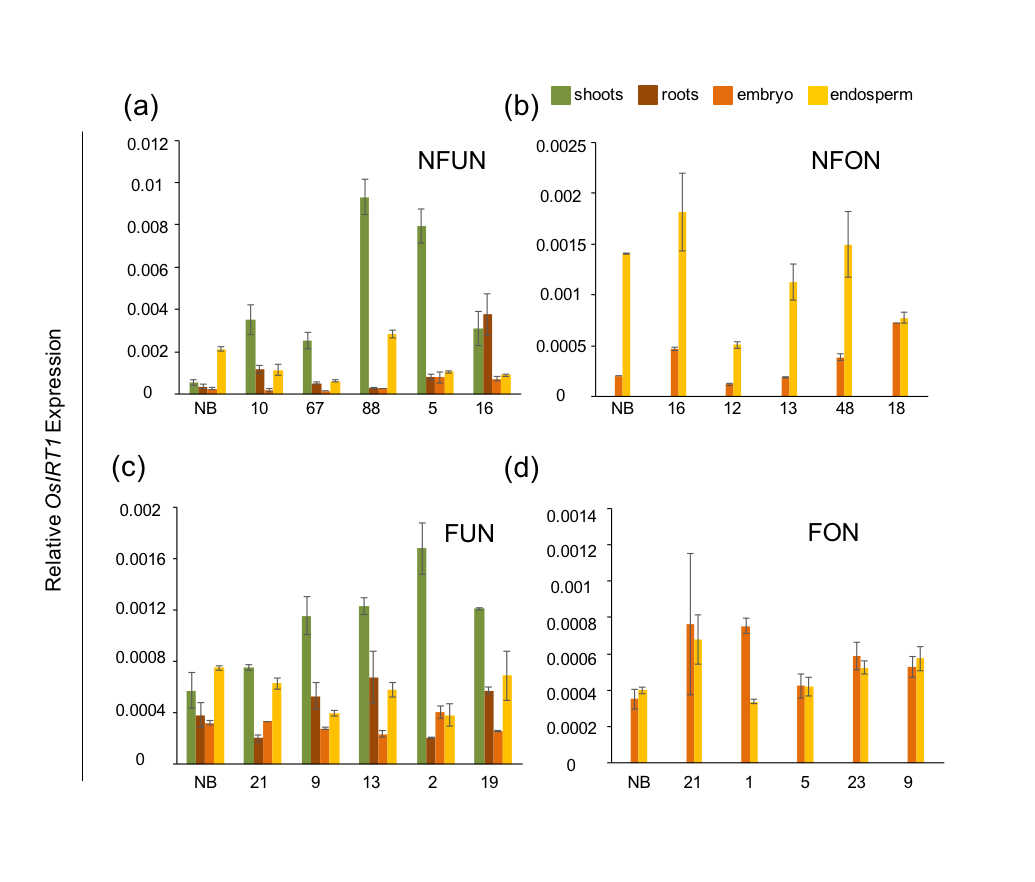
**

**Figure S6 Expression analysis of iron homeostasis related genes.** Expression of selected endogenous genes was detected by qRT-PCR in shoots and roots of (a) FUN and FON plants and (b) NFUN and NFON plants, subjected to iron sufficient and iron deficient growth conditions. These studied genes included those encoding enzymes for NA and DMA synthesis (*OsNAS1*, *OsNAAT1*), transcription factors (*OsIDEF1*, *OsIRO2*), iron-regulated transporters and intercellular transporters (*OsIRT1*, *OsNRAMP1*, *OsYSL2*, *OsFRO1*), and FERRITIN (*OsFER1*). See Figure 1a for the abbreviation of the gene cassettes.

**
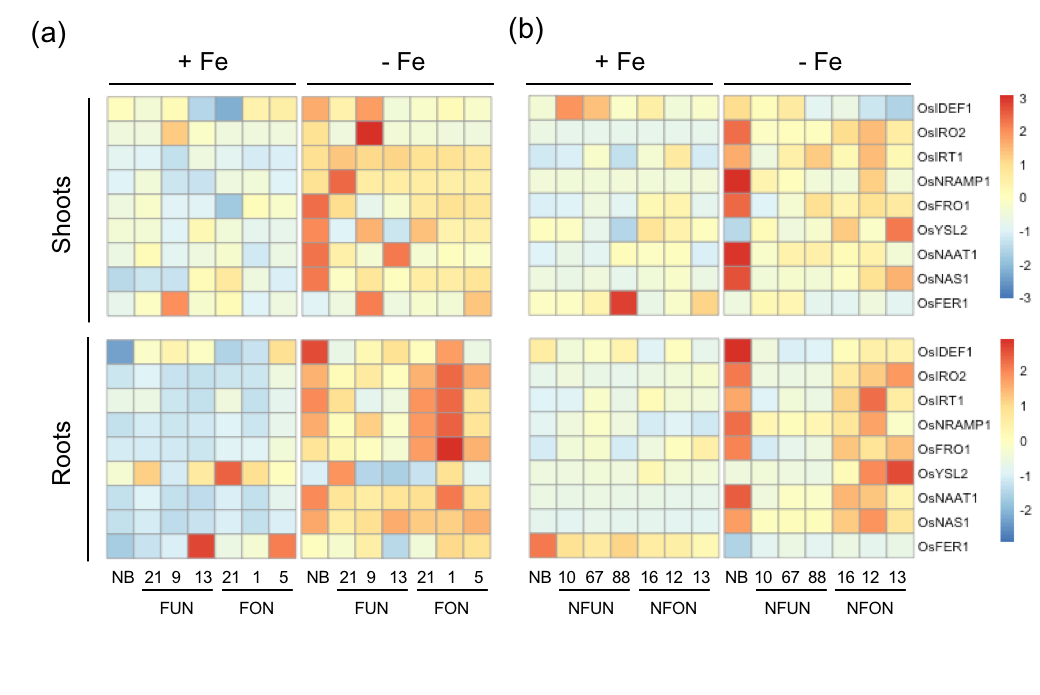
**

**Figure S7 Characterization of IR64 transgenic lines in T0 generation using southern hybridization and iron concentration in the polished T1 grains in the single insertion lines.** (a) Southern hybridization analysis using PMI as a probe. Numbers indicate independent transformed lines with potential single insertion of transgene. (b) The bars for each line represent three individual plants. Values are the average of two technical replicates. Arrows indicate the selected plants from each line for further analysis in next generation.

**
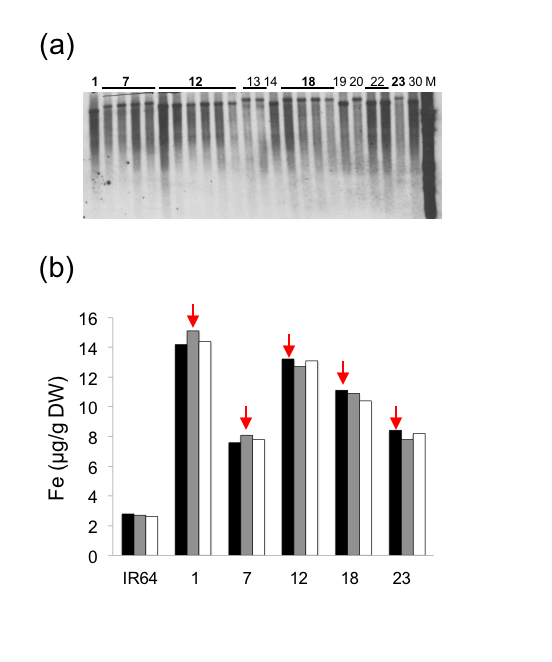
**

**Table S1 Phenotypic assessment of selected transgenic lines in T2 generation grown in the greenhouse condition.** Parameters including days to flowering (DTF), soil-plant analysis development (SPAD) value, plant height, tiller number, one thousand grain weight (1000grain weight), panicle weight and panicle filling rate are presented. Values are the average of three individual plants. Panicle weight and panicle filling rate are the average of 12 panicles per line (T4 grains). Black and red asterisks indicate statistically significantly higher and lower values as compared to the NB control (*p<0.05, **p<0.01), respectively. The number that follows the ± sign is the standard deviation (SD). See Figure 1a for the abbreviation of the gene cassettes.

| **Plant line** | **Height (cm)** | **Tiller No.** | **SPAD** | **DTF** | **1000GrainWeight (g)** | **Panicle weight (g)** | **Grain filling (%)** |
| --- | --- | --- | --- | --- | --- | --- | --- |
| NB | 63.33 (±2.08) | 7.67 (±1.53) | 36.00 (±3.00) | 81.00 (±1.00) | 24.17 (±0.29) | 4.49 (±0.89) | 79.28 (±1.44) |
| **NFUN** |  |  |  |  |  |  |  |
| 5 | 59.00 (±2.00) | 7.67 (±1.53) | 38.00 (±1.00) | 79.33 (±1.53) | 25.83 (±0.76) | 4.48 (±0.76) | 83.88 (±1.41)* |
| 67 | 61.67 (±2.31) | 12.00 (±2.00)* | 36.00 (±1.00) | 78.33 (±1.53) | 22.50 (±0.87)* | 4.27 (±0.54) | 82.53 (±1.09) |
| 88 | 63.00 (±2.00) | 11.33 (±2.08)* | 38.33 (±1.53) | 84.00 (±1.00) | 25.33 (±0.29) | 4.35 (±0.57) | 72.01 (±1.47)* |
| 26 | 59.00 (±2.00) | 9.00 (±1.00) | 38.33 (±2.08) | 79.00 (±1.00) | 26.17 (±0.29) | 4.51 (±0.54) | 85.03 (±1.11) |
| 16 | 54.33 (±1.53)* | 8.33 (±0.58) | 39.00 (±1.00) | 81.33 (±0.58) | 25.33 (±0.76) | 4.42 (±0.57) | 83.29 (±0.98)* |
| 10 | 60.67 (±1.53) | 6.67 (±0.58) | 38.33 (±1.15) | 82.00 (±1.00) | 24.33 (±0.29) | 4.35 (±0.65) | 80.85 (±2.01) |
| **NFON** |  |  |  |  |  |  |  |
| 7 | 58.33 (±1.15) | 10.67 (±2.08)* | 37.00 (±2.00) | 78.00 (±1.00) | 23.00 (±0.50) | 4.65 (±0.92) | 84.07 (±1.08)* |
| 16 | 55.00 (±2.00)* | 9.00 (±1.00) | 36.67 (±2.08) | 80.00 (±1.00) | 24.00 (±0.50) | 4.54 (±0.88) | 80.04 (±1.10) |
| 48 | 59.33 (±1.53) | 12.00 (±1.00)* | 36.67 (±0.58) | 82.67 (±3.21) | 23.67 (±0.76) | 4.53 (±0.79) | 81.60 (±1.96) |
| 13 | 62.67 (±1.53) | 12.00 (±2.00)* | 36.67 (±2.08) | 79.00 (±2.00) | 24.50 (±0.50) | 4.62 (±0.89) | 82.17 (±0.89) |
| 18 | 58.33 (±1.53) | 7.33 (±1.53) | 38.00 (±1.00) | 81.00 (±1.00) | 23.67 (±0.58) | 4.57 (±0.76) | 83.26 (±0.98)* |
| 12 | 61.50 (±1.40) | 10.00 (±1.20)* | 39.00 (±2.00) | 83.00 (±1.00) | 23.70 (±0.58) | 4.48 (±0.45) | 81.97 (±1.15) |
| **FUN** |  |  |  |  |  |  |  |
| 2 | 61.33 (±2.08) | 8.33 (±2.08) | 38.67 (±1.53) | 83.67 (±1.53) | 25.00 (±0.50) | 4.30 (±0.66) | 79.02 (±1.11) |
| 6 | 59.00 (±2.00) | 7.33 (±1.15) | 36.33 (±1.53) | 80.00 (±1.00) | 25.50 (±0.50) | 4.77 (±1.07) | 81.10 (±1.10) |
| 16 | 61.00 (±1.00) | 7.33 (±0.58) | 38.67 (±1.53) | 78.67 (±1.53) | 22.83 (±0.58) | 4.42 (±0.54) | 79.83 (±1.21) |
| 19 | 58.33 (±1.53) | 7.00 (±1.00) | 38.33 (±1.53) | 82.33 (±1.53) | 23.50 (±0.50) | 4.62 (±0.67) | 82.46 (±1.01) |
| 9 | 59.67 (±1.53) | 8.33 (±1.15) | 37.86 (±0.58) | 82.67 (±3.21) | 24.86 (±0.50) | 4.42 (±0.88) | 80.16 (±1.21) |
| 10 | 60.00 (±1.00) | 11.33 (±1.00)* | 36.67 (±1.08) | 82.33 (±1.53) | 23.12 (±0.58) | 4.56 (±0.68) | 85.84 (±1.03)* |
| **FON** |  | | | | |  |  |
| 21 | 59.33 (±1.00) | 7.67 (±1.00) | 38.67 (±1.08) | 79.00 (±1.53) | 22.85 (±0.58) | 4.83 (±0.99)* | 80.37 (±1.11) |
| 1 | 58.33 (±0.53) | 8.33 (±1.15) | 37.33 (±0.58) | 80.00 (±1.00) | 24.12 (±0.50) | 4.53 (±0.84) | 83.19 (±1.08)* |
| 5 | 57.67 (±1.53)* | 7.33 (±0.58) | 39.33 (±2.00) | 78.00 (±1.00) | 23.50 (±0.76) | 4.48 (±0.57) | 81.03 (±1.10) |
| 9 | 59.67 (±1.53) | 9.83 (±1.15)* | 39.00 (±1.00) | 78.67 (±1.53) | 22.85 (±0.58) | 4.75 (±0.91) | 79.63 (±0.97) |
| 23 | 58.67 (±1.53) | 9.83 (±1.15)* | 36.67 (±2.08) | 81.00 (±1.50) | 23.50 (±0.28) | 4.68 (±0.89) | 82.05 (±1.13) |

| **Line** | **Length (mm)** | **Width (mm)** | **Chalkiness (%)** | **Amylose content (%)** | **Starch content (%)** | **Protein content (%)** |
| --- | --- | --- | --- | --- | --- | --- |
| NB | 5.63 (±0.12) | 2.9 (±0.17) | 1.5 (±0.3) | 17.24 (±0.38) | 83.42 (±0.75) | 10.3 (±0.07) |
| **NFUN** |  |  |  |  |  |  |
| 10 | 5.54 (±0.14) | 2.91 (±0.14) | 1.2 (±0.3) | 18.08 (±1.31) | 83.57 (±0.44) | 10.6 (±0.08) |
| 67 | 5.46 (±0.14) | 2.89 (±0.14) | 1 (±0.2) | 17.21 (±1.29) | 81.87 (±0.69)* | 10.1 (±0.09) |
| 88 | 5.24 (±0.15) | 2.7 (±0.16) | 1.3 (±0.2) | 19.29 (±1.44) | 84.03 (±0.87) | 11.1 (±0.14) |
| **NFON** |  |  |  |  |  |  |
| 16 | 5.48 (±0.12) | 2.93 (±0.09) | 2 (±0.3) | 19.57 (±1.24) | 82.19 (±0.73) | 10.7 (±0.07) |
| 12 | 5.39 (±0.12) | 2.83 (±0.12) | 1 (±0.2) | 18.86 (±0.75) | 81.73 (±0.98)* | 10.3 (±0.07) |
| 13 | 5.48 (±0.12) | 2.88 (±0.10) | 0.8 (±0.1)* | 17.95 (±0.98) | 83.03 (±1.03) | 10.4 (±0.06) |
| **FUN** |  |  |  |  |  |  |
| 21 | 5.16 (±0.14)* | 2.6 (±0.12)* | 0.8 (±0.1)* | 18.53 (±0.76) | 82.69 (±1.12) | 10.2 (±0.12) |
| 9 | 5.44 (±0.10) | 2.87 (±0.14) | 0.8 (±0.08)* | 17.44 (±1.32) | 81.96 (±0.57)* | 10.8 (±0.10) |
| 13 | 5.45 (±0.11) | 2.87 (±0.12) | 0.5 (±0.1)* | 17.85 (±1.21) | 82.43 (±0.64) | 10.7 (±0.11) |
| **FON** |  |  |  |  |  |  |
| 21 | 5.44 (±0.06) | 2.93 (±0.11) | 0.4 (±0.1)* | 17.89 (±1.13) | 81.93 (±0.73)* | 10.6 (±0.14) |
| 1 | 5.48 (±0.09) | 2.93 (±0.08) | 2.2 (±0.3)* | 17.54 (±1.23) | 83.03 (±0.63) | 10.3 (±0.08) |
| 5 | 5.49 (±0.07) | 2.92 (±0.10) | 0.7 (±0.2)* | 17.28 (±1.17) | 82.47 (±0.48) | 10.3 (±0.07) |

**Table S2 Grain quality assessment of selected transformed plants (T3) carrying NFUN, FNON, FUN and FON constructs as compared to the Nipponbare control**.

| **Plant line** | **Plant Height (cm)** | **Tiller No.** | **DTF** | **Grain Width (cm)** | **Grain Length (cm)** | **1000grain Weight (g)** | **Grain Filling (%)** |
| --- | --- | --- | --- | --- | --- | --- | --- |
| IR64 | 82.67 (±2.52) | 11.00 (±1.00) | 115.33 (±2.52) | 0.190 (±0.02) | 0.761 (±0.05) | 21.8 (±0.81) | 85.00 (±2.00) |
| 1 | 86.00 (±2.65) | 8.00 (±1.00)* | 117.00 (±3.61) | 0.199 (±0.01) | 0.756 (±0.05) | 22.3 (±0.81) | 82.03 (±0.45) |
| 12 | 84.33 (±3.79) | 12.00 (±2.65) | 119.67 (±0.58) | 0.184 (±0.02) * | 0.658 (±0.04) * | 15.7 (±0.58) * | 79.33 (±4.04) * |
| 18 | 83.33 (±1.53) | 11.67 (±2.08) | 116.67 (±1.53) | 0.197 (±0.01) | 0.800 (±0.06) | 21.3 (±0.40) | 81.93 (±5.37) |
| 23 | 86.67 (±3.51) | 11.33 (±1.53) | 117.00 (±1.00) | 0.199 (±0.01) | 0.758 (±0.07) | 21.7 (±0.93) | 83.67 (±3.21) |
| 7 | 84.33 (±5.13) | 9.67 (±2.08) | 117.33 (±2.52) | 0.198 (±0.03) | 0.741 (±0.04) | 21.3 (±0.75) | 84.67 (±2.52) |

**Table S3 Phenotypic assessment of selected transgenic lines (cv. IR64 background) from the T1 generation grown in greenhouse conditions.**

| **Primer Name** | **Direction** | **Primer sequence (5’-3’)** | **Purpose** |
| --- | --- | --- | --- |
| Ubi-mut-1 | F | tcctctagagtcgaccttcagaagtaacaccaaac | cloning |
|  | R | gtttggtgttacttctgaaggtcgactctagagga |  |
| AtNRAMP-F-BamHI | F | ggatccatgccacaactcgagaac | cloning/ sequencing |
| AtNRAMP-R-BspEI | R | tccggatcaatgactagactccg |  |
| Ole18-f-PstI-SphI | F | gcatgcctgcaggcaaagtattttggc | cloning/ sequencing |
| Ole18-BamHI | R | aaggatccgaactcttgtttgatggtgtttg |  |
| PvFer | F | gtcttcgtttatatttgctcttg | PCR/ sequencing |
| Ubi | R | gtaaatcccaaatcccaattacc | PCR/ sequencing |
| 35S | F | cagttcatacagagtctcttacg | PCR/ sequencing |
| PMI | F | ctggctaatggtggtttct | PCR/ southern probe |
|  | R | cgtgatgtgattgagagt |  |
| AtNRAMP3-1 | F | gtgttagttaaccattagctgtg | PCR/ sequencing |
|  | R | attagctaaattacaattagaac |  |
| AtNRAMP3-2 | F | gatcaatctgtttgtcacaactg | PCR/ sequencing |
|  | R | cgatcgcccaaatgtatagtatc |  |
| qAtNRAMP3 | F | gatcaatctgtttgtcacaactg | qRT-PCR |
|  | R | cgatcgcccaaatgtatagtatc |  |
| qAtNAS1 | F | gcacttggagaaacacatgg | qRT-PCR |
|  | R | tctgagagcatgagcactcc |  |
| qPvFER | F | ggggatacgggaaaacgtaa | qRT-PCR |
|  | R | gagcctggctaaccgtcat |  |
| OsIDEF1 | F | gtcttcaggctggggatgt | qRT-PCR |
|  | R | gggatttgttgtctgctgatg |  |
| OsIRO2 | F | gaaggtcttcacttcatcagttca | qRT-PCR |
|  | R | tgatcgttccttcacttctctg |  |
| OsIRT1 | F | ctcgagataggcatcgtggt | qRT-PCR |
|  | R | gaacatctggtggaagcaca |  |
| OsNRAMP1 | F | ggaaggtggtggacgaca | qRT-PCR |
|  | R | ggtccaatgtgggacaaaaa |  |
| OsFRO1 | F | tcgccataccacctgatgta | qRT-PCR |
|  | R | ttgccttcctcgatcctatg |  |
| OsYSL2 | F | gggctccttaacttgcttcc | qRT-PCR |
|  | R | gaggggtatggaatccgttt |  |
| OsNAAT1 | F | tggagggaatccatgatga | qRT-PCR |
|  | R | cttcattcccagcacactcc |  |
| OsNAS1 | F | cggttgagaaggcagaagagt | qRT-PCR |
|  | R | cgatcgtccggctgttag |  |
| OsFER1 | F | ggattcgccaaattcttcaa | qRT-PCR |
|  | R | cctttctcaggatggtcgaa |  |
| OsUBQ5 | F | accacttcgaccgccactact | qRT-PCR |
|  | R | acgcctaagcctgctggtt |  |

**Table S4 Primer sequences used for sequence validation, cloning and qRT-PCR analysis**

**Table S5 Primer sequences used for TAIL-PCR analysis on NFON16**

| Primer Name | Primer Sequence (5’ – 3’) | Purpose |
| --- | --- | --- |
| AD1 | NGT CGA SWG ANA WGA A | TAIL-PCR |
| AD2 | GTN CGA SWC ANA WGT T | TAIL-PCR |
| AD3 | AGW GNA GWA NCA WAG G | TAIL-PCR |
| ON-SP1 | TTG GCC GTT CAA AGC GGA GTC TAG TCA T | TAIL-PCR |
| ON-SP2 | TGG TGC ATT CAT CCT CTA CCT CAT TGC TC | TAIL-PCR |
| ON-SP3 | TCA TGG GTA GCT TCA AGA TTG GTC CTT TG | TAIL-PCR |
| ON-SP4 | CTT GTT GCT GCG CTC GTG ATA ATG ATC | TAIL-PCR |
| ON-SP5 | ACC CAA CTT AAT CGC CTT GCA GCA CAT CCC | TAIL-PCR |
| ON-SP6 | CAC CGA TCG CCC TTC CCA ACA GTT G | TAIL-PCR |
| ON-SP7 | AGC TTG GAT CAG ATT GTC GTT TCC | Sequencing (right border) |
| ON-LP1 | GAT GTT CCT GTT CGC TGA AAC ACC GCA | TAIL-PCR |
| ON-LP2 | CAG TGG ATG ATT TTG CCT TCT CGC TGC A | TAIL-PCR |
| ON-LP3 | CGT TGT GGA AAG GTT CTC AGC AGT TAC AGC | TAIL-PCR |
| TL1 | TAG GGT TCC TAT AGG GTT TCG CTC A | TAIL-PCR |
| TL2 | GTG TTG AGC ATA TAA GAA ACC CTT AG | TAIL-PCR |
| TL3 | CCT AAA ACC AAA ATC CAG | TAIL-PCR |
| TL4 | CCC GAA TTA ATT CGG CGT | Sequencing (left border) |
